# Perfil Aterosclerótico da Artéria Carótida como Marcador de Progressão para Doença Cardiovascular

**DOI:** 10.36660/abc.20210229

**Published:** 2021-04-08

**Affiliations:** 1 Faculdade de Ciências Médicas da Santa Casa de São Paulo São PauloSP Brasil Faculdade de Ciências Médicas da Santa Casa de São Paulo, São Paulo, SP - Brasil; 2 Universidade de São Paulo Faculdade de Medicina Instituto de Radiologia do Hospital das Clínicas São PauloSP Brasil Instituto de Radiologia do Hospital das Clínicas (InRad) - Faculdade de Medicina, Universidade de São Paulo, São Paulo, SP - Brasil

**Keywords:** Doenças Cardiovasculares, Aterosclerose, Mortalidade, Doença Arterial Coronariana, Acidente Vascular Cerebral, Placa Aterosclerótica

As doenças diretamente ligadas à aterosclerose estão entre as principais causas de mortalidade em todo o mundo, com destaque para o acidente vascular cerebral isquêmico e a doença arterial coronariana (DAC).^1^ O diagnóstico de doença cardiovascular ou sua progressão em seus estágios iniciais, com o objetivo de aplicar medidas que possam prevenir ou retardar sua progressão e complicações subsequentes é atualmente um grande desafio.^2^ A identificação e caracterização das placas ateroscleróticas permitem identificar um número significativo de pacientes com escores de risco baixo ou intermediário.^2^ Muitos desses pacientes não seriam identificados pelos algoritmos disponíveis para doenças cardiovasculares, o que poderia resultar na falta do manejo correto.

A quantificação da placa da artéria carótida pode ser uma medida de aterosclerose que deve estar associada ao futuro risco de doença cardiovascular aterosclerótica, abrangendo doenças coronárias, cerebrovasculares e arteriais periféricas.^3^ Sabe-se que a função endotelial deficiente e o aumento da espessura da camada íntima da carótida são eventos substanciais no processo aterosclerótico,^4^^,^^5^ com imagens de características da carótida sendo utilizadas para prever o risco de eventos cardiovasculares.^3^

A aterosclerose é um processo inflamatório difuso que afeta a camada íntima arterial com extensa deposição de lípides, formação de células espumosas e migração de células do músculo liso vascular.^6^ A placa resultante pode causar sintomas devido ao estreitamento progressivo dos vasos ou migração de pequenos fragmentos.

O diagnóstico da placa aterosclerótica carotídea mudou da quantificação da estenose pura para a caracterização da placa, o que permite uma melhor compreensão fisiopatológica e uma estratificação de risco e manejo do paciente mais precisos.^3^ A medida da espessura da camada íntima-média (CIM) e do escore da placa (EP) - ambos utilizando ultrassonografia de carótida - fornecem informações sobre a extensão do dano vascular estrutural,^5^ refletindo um possível envolvimento cardíaco coronário.

Assim, o uso de ultrassom para a detecção e avaliação da aterosclerose, particularmente através da avaliação da placa carotídea e, mais recentemente, a avaliação da placa femoral, está se tornando cada vez mais utilizado na tomada de decisão clínica para pacientes com DAC prevalente e em risco.^7^ Entretanto, pesquisas limitadas baseadas em desfechos confirmaram a associação entre a carga da placa carotídea avaliada por ultrassom e eventos cardiovasculares.^7^ Representando um comprometimento sistêmico, o estudo das placas carotídeas fornece informações indiretas sobre o perfil aterosclerótico e também sobre o maior ou menor risco associado à DAC.^6^

No artigo “Perfil aterosclerótico da artéria carótida como preditor de risco para reestenose após implante de stent coronário”, os autores vão além da utilização usual de imagens da carótida para o rastreamento de risco cardiovascular.^8^ Ao avaliar mais de 100 pacientes submetidos à intervenção coronária percutânea, eles correlacionam a presença de placas ateroscleróticas ecolucentes na artéria carótida com um risco aumentado de reestenose coronária intra-stent. Esse achado pode introduzir uma nova ferramenta no seguimento de pacientes com DAC e, talvez, influenciar na decisão quanto ao tipo de stent a ser implantado na angioplastia coronariana.
