# Supplementary material for: Perfil Aterosclerótico da Artéria Carótida como Marcador de Progressão para Doença Cardiovascular
Source: Arq Bras Cardiol. 2021 Apr 8;116(4):734–5. [Article in Portuguese] doi: 10.36660/abc.20210229 (PMC8121387; doi:10.36660/abc.20210229)
Supplement: Translation

# Translation: Carotid Artery Atherosclerotic Profile as a Progression Marker for Cardiovascular Disease

**Affiliations:** 1 São PauloSP Brasil Faculdade de Ciências Médicas da Santa Casa de São Paulo, São Paulo, SP - Brasil; 2 São PauloSP Brazil Instituto de Radiologia do Hospital das Clínicas (InRad) - Faculdade de Medicina, Universidade de São Paulo, São Paulo, SP - Brazil

**Keywords:** Cardiovascular Diseases, Atherosclerosis, Mortality, Coronary Artery Disease, Stroke, Plaque, Atherosclerosis

Conditions directly linked to atherosclerosis are among the main causes of mortality worldwide, notably ischemic stroke and coronary artery disease (CAD).^1^ Diagnosing cardiovascular disease or its progression in the early stages, aiming to apply measures that can prevent or delay its progression and subsequent complications is currently a major challenge.^2^ The identification and characterization of atherosclerotic plaques allows the identification of a significant number of patients with low or intermediate risk scores.^2^ Many of these patients would not be identified by the available algorithms for cardiovascular diseases, which could result in the lack of correct management.

The quantification of the carotid artery plaque may be a measure of atherosclerosis, which should be associated with future risk of atherosclerotic cardiovascular disease, encompassing coronary, cerebrovascular, and peripheral arterial diseases.^3^ It is already known that impaired endothelial function and increased carotid intima-media thickness are substantial events in the atherosclerotic process,^4^^,^^5^ with imaging of carotid features being used to predict the risk of cardiovascular events.^3^

Atherosclerosis is a diffuse inflammatory process that affects the arterial intima with extensive lipid deposition, foam cell formation, and vascular smooth muscle cell migration.^6^ The resulting plaque can cause symptoms due to progressive vessel narrowing or small fragment migration.

The diagnosis of carotid atherosclerotic plaque has shifted from pure stenosis quantification to plaque characterization, which allows an improved pathophysiological understanding, and for more precise patient risk stratification and management.^3^ Measurement of the intima-media thickness (IMT) and the plaque score (PS) – both using carotid ultrasonography - provide information on the extent of structural vascular damage^5^ reflecting a possible coronary heart involvement.

Thus, using ultrasound for the detection and evaluation of atherosclerosis, particularly through carotid plaque assessment, and more recently, femoral plaque assessment, is becoming increasingly utilized in clinical decision-making for both at-risk and prevalent CAD patients.^7^ Nevertheless, limited outcome-based research has confirmed the association between ultrasound-assessed carotid plaque burden and cardiovascular events.^7^

Representing a systemic impairment, the study of carotid plaques provides indirect information about the atherosclerotic profile and also about the greater or lesser risk associated with CAD.^6^

In the article “Carotid artery atherosclerotic profile as risk predictor for restenosis after coronary stenting” the authors go beyond the usual use of carotid images for cardiovascular risk tracking.^8^ By evaluating more than 100 patients undergoing percutaneous coronary intervention, they correlate the presence of echolucent atherosclerotic plaques in the carotid artery with an increased risk of coronary in-stent restenosis. This finding may introduce a new tool in the follow-up of CAD patients and, maybe, influence the decision regarding the type of stent to be implanted in coronary angioplasty.
